# Supplementary figures and images for: Procyclic trypanosomes recycle glucose catabolites and TCA cycle intermediates to stimulate growth in the presence of physiological amounts of proline
Source: PLoS Pathog. 2021 Mar 1;17(3):e1009204. doi: 10.1371/journal.ppat.1009204 (PMC7951978; doi:10.1371/journal.ppat.1009204)

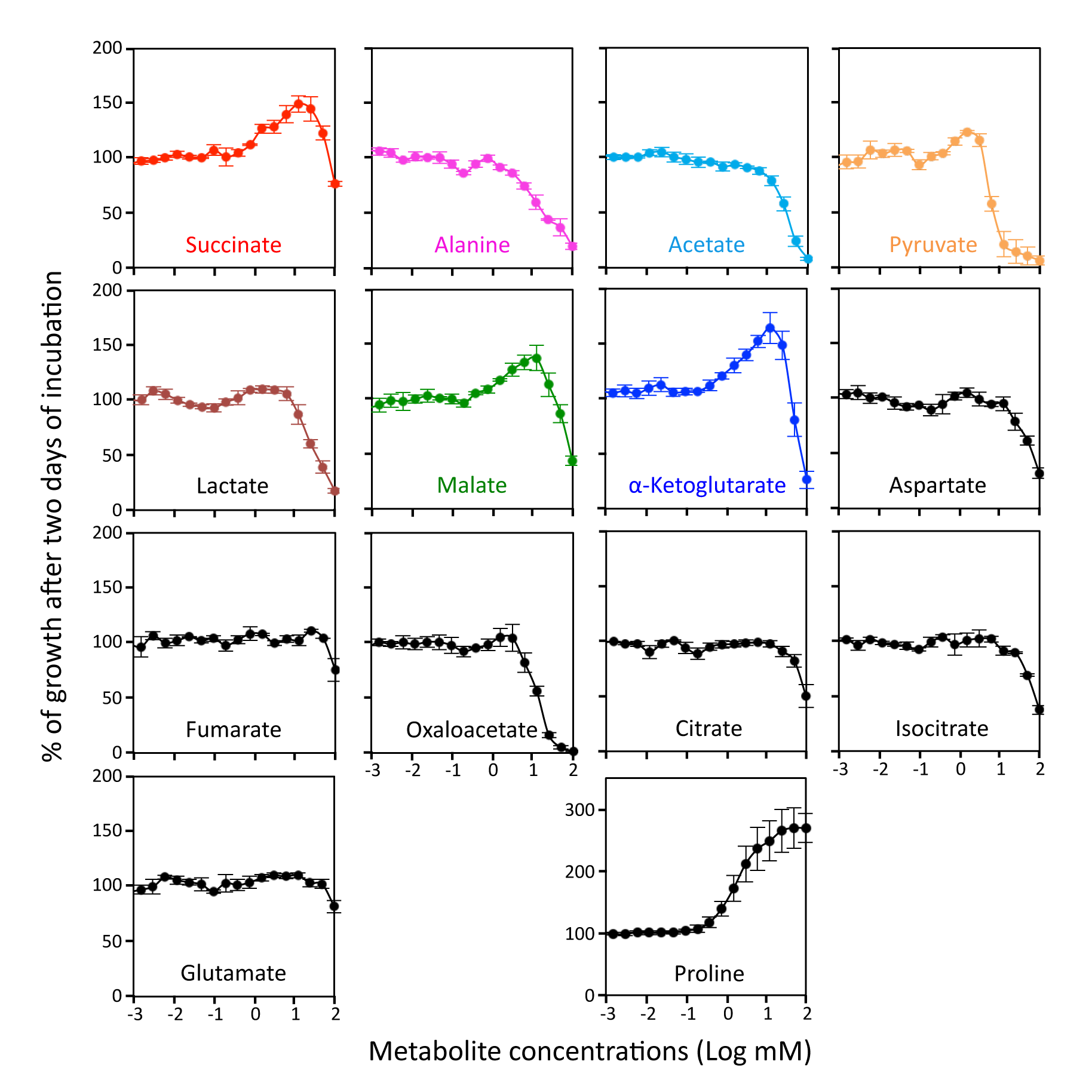

Supplement: S1 Fig — Incubation was started at 2 x 10^6^ cell density and the Alamar Blue assay was performed after 48 h at 27°C as described before [23]. (TIF) [file ppat.1009204.s001.tif]

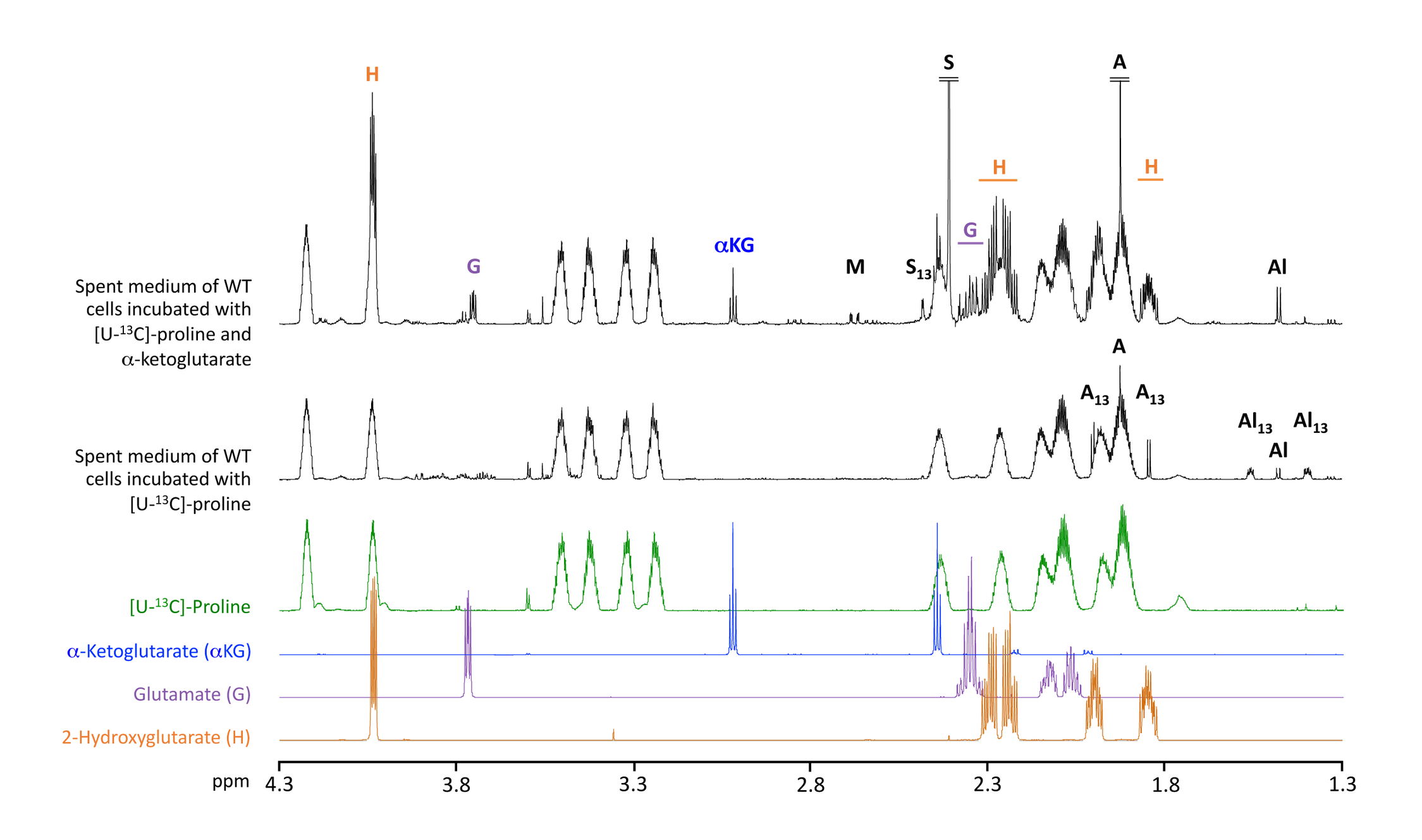

Supplement: S2 Fig — 1H-NMR analysis of samples (black) and controls (colored) performed at 800 MHz to identify acetate (A), alanine (Al), glutamate (G), 2-hydroxyglutarate (H), α-ketoglutarate (αKG), malate (M), proline and succinate (S). The resonances corresponding to 13C-enriched molecules are indicated in index. (TIF) [file ppat.1009204.s002.tif]

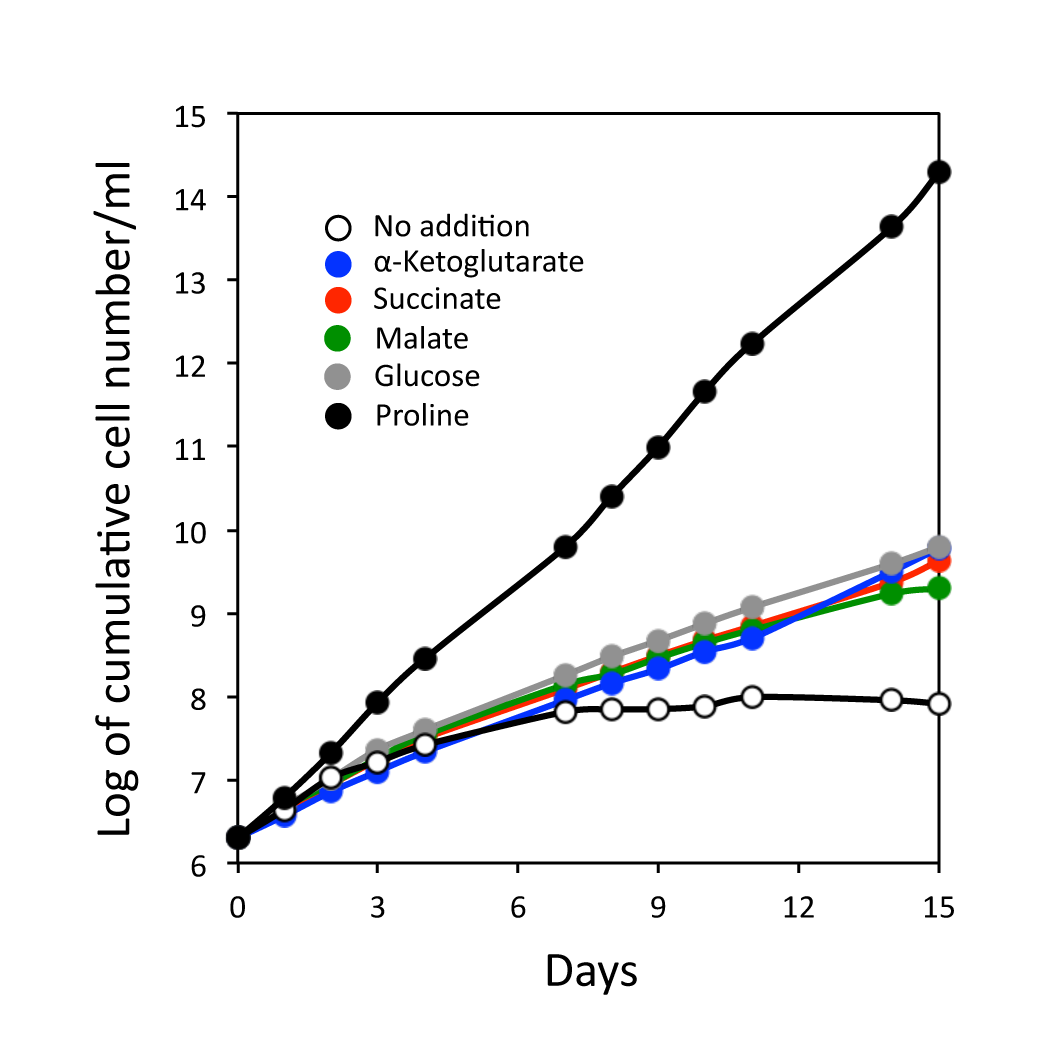

Supplement: S3 Fig — Cells were maintained in the exponential growth phase (between 106 and 107 cells/ml), and cumulative cell numbers reflect normalization for dilution during cultivation. (TIF) [file ppat.1009204.s003.tif]

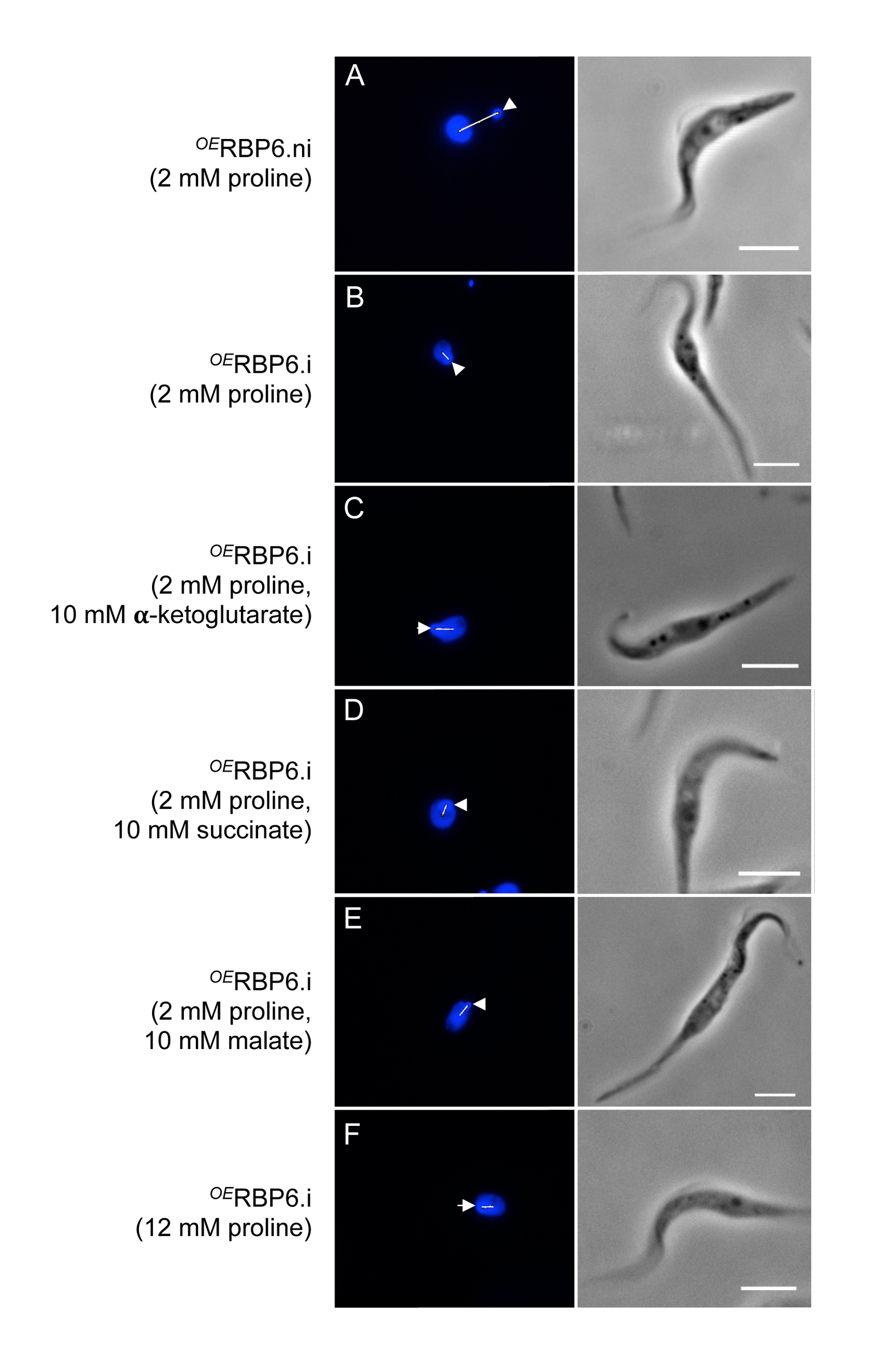

Supplement: S4 Fig — Illustration of microscopic analyses of non-induced (.ni) (A) or induced (.i) (B-F) OERBP6 cells grown in the presence of 2 mM proline complemented or not (A-B) with 10 mM of α-ketoglutarate (C), succinate (D), malate (E) or proline (F). The non-induced population is composed of procyclic trypanosomes (A), while epimastigote-like cells mainly composed the induced population regardless of the culture conditions. DAPI staining of DNA is shown on the left panels, in which kinetoplasts are highlighted by arrowheads and the distance between kinetoplasts and nuclei are shown by white lines, while the right panels show phase contrast (calibration bar: 5 μm). These analyses were performed three days post induction (B-F). (TIF) [file ppat.1009204.s004.tif]

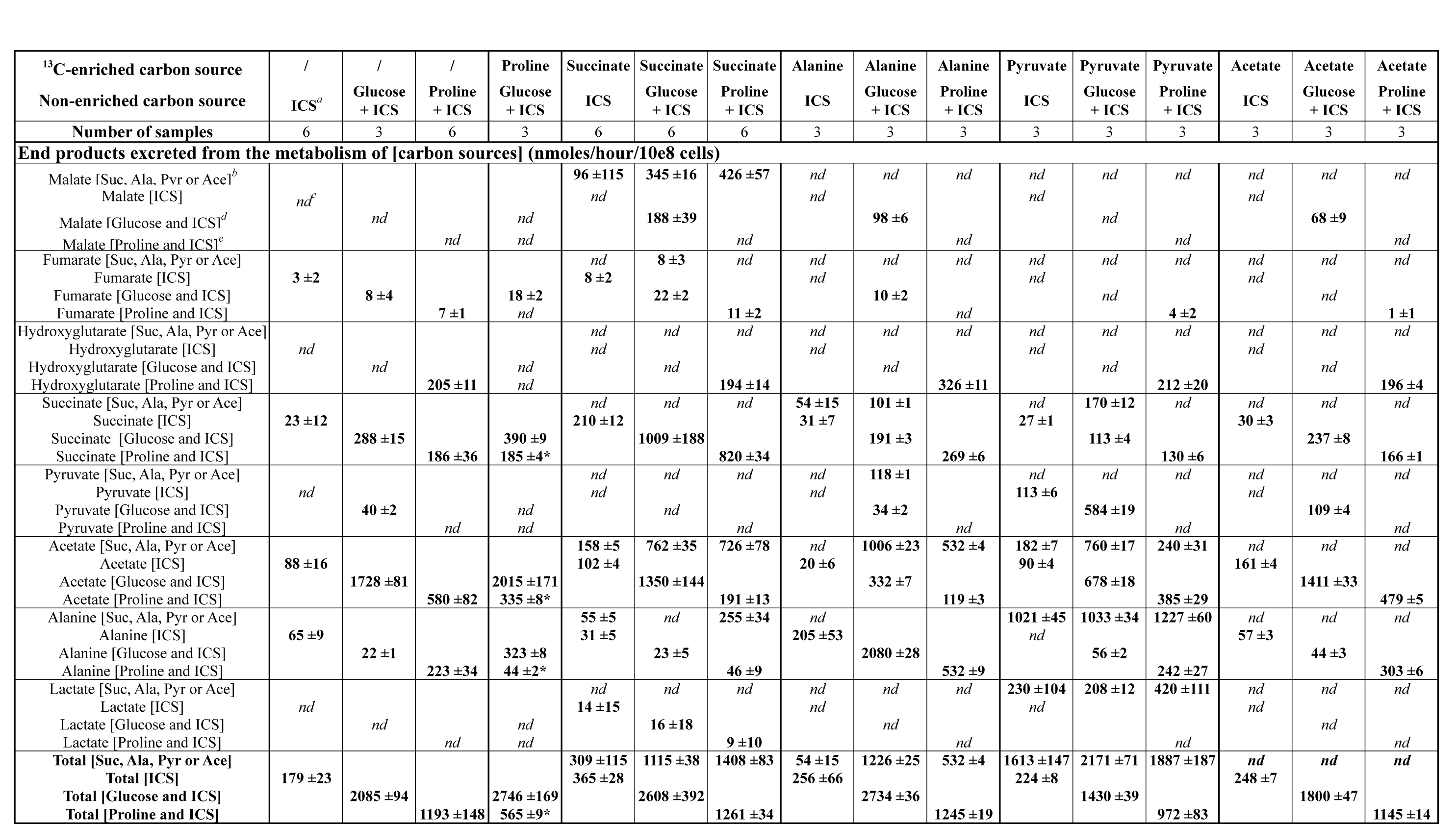

Supplement: S1 Table — The parasites were incubated with 4 mM [U-13C]-succinate, [U-13C]-alanine, [U-13C]-pyruvate or [U-13C]-acetate in the presence or absence of 4 mM glucose or proline. a ICS (internal carbon source): intracellular carbon source of unknown origin metabolized by the PCF trypanosomes. b Amounts of end-products excreted (here malate) from the carbon source indicated in brackets, expressed as nmoles excreted per h and per 108 cells. c nd: not detectable. d End-products excreted (here malate) from glucose or the ICS, which are both non-enriched. e End-products excreted (here malate) from proline or the ICS, which are both non-enriched. The asterisks mean that in this particular experiment the values correspond to proline only, since it is 13C-enriched. (TIF) [file ppat.1009204.s005.tif]
